# Particulate cytoplasmic structures with high concentration of ubiquitin-proteasome accumulate in myeloid neoplasms

**DOI:** 10.1186/s13045-015-0169-6

**Published:** 2015-06-18

**Authors:** Alessandro Pecci, Vittorio Necchi, Serena Barozzi, Agostina Vitali, Emanuela Boveri, Chiara Elena, Paolo Bernasconi, Patrizia Noris, Enrico Solcia

**Affiliations:** Department of Internal Medicine, IRCCS Policlinico San Matteo Foundation and University of Pavia, Pavia, Italy; Department of Molecular Medicine, University of Pavia, Pavia, Italy; Centro Grandi Strumenti, University of Pavia, Pavia, Italy; Pathologic Anatomy Section, Department of Diagnostic Medicine, IRCCS Policlinico San Matteo Foundation, Pavia, Italy; Hematology Section, Department of Oncology and Hematology, IRCCS Policlinico San Matteo Foundation, Pavia, Italy

**Keywords:** Ubiquitin/proteasome system, Proteasome, Polyubiquitinated proteins, Chaperone molecules, Myeloid neoplasia, Myeloproliferative neoplasms

## Abstract

**Background:**

Increased plasma levels of proteasome have been associated with various neoplasms, especially myeloid malignancies. Little is known of the cellular origin and release mechanisms of such proteasome. We recently identified and characterized a novel particulate cytoplasmic structure (PaCS) showing selective accumulation of ubiquitin-proteasome system (UPS) components. PaCSs have been reported in some epithelial neoplasms and in two genetic disorders characterized by hematopoietic cell dysplasia and increased risk of leukemia. However, no information is available about PaCSs in hematopoietic neoplasms.

**Methods:**

PaCSs were investigated by ultrastructural, immunogold, and immunofluorescence analysis of bone marrow (BM) biopsies and peripheral blood (PB) cell preparations of 33 consecutive, untreated, or relapsed patients affected by different hematopoietic neoplasms. BM and PB samples from individuals with non-neoplastic BM or healthy donors were studied as controls. Granulocytes and platelet proteasome content was measured by immunoblotting and plasma proteasome levels by ELISA.

**Results:**

PaCSs with typical, selective immunoreactivity for polyubiquitinated proteins and proteasome were widespread in granulocytic cells, megakaryocytes, and platelets of patients with myeloproliferative neoplasms (MPN). In acute myeloid leukemia and myelodysplastic syndromes (MDS), PaCSs were only occasionally detected in blast cells and were found consistently in cells showing granulocytic and megakaryocytic maturation. Conversely, PaCSs were poorly represented or absent in non-neoplastic hematopoietic tissue or lymphoid neoplasms. In MPN granulocytes and platelets, the presence of PaCSs was associated with increased amounts of proteasome in cell lysates. PaCSs were often localized in cytoplasmic blebs generating PaCSs-filled plasma membrane vesicles observable in the BM intercellular space. In MPN and MDS, accumulation of PaCSs was associated with significant increase in plasma proteasome. Immunogold analysis showed that PaCSs of myeloid neoplasia selectively concentrated the chaperone proteins Hsp40, Hsp70, and Hsp90.

**Conclusions:**

PaCSs accumulate in cells of myeloid neoplasms in a lineage- and maturation-restricted manner; in particular, they are widespread in granulocytic and megakaryocytic lineages of MPN patients. PaCSs development was associated with excess accumulation of polyubiquitinated proteins, proteasome, and chaperone molecules, indicating impairment of the UPS-dependent protein homeostasis and a possible link with Hsp90-related leukemogenesis. A mechanism of PaCSs discharge by leukemic cells could contribute to increased plasma proteasome of MPN and MDS.

**Electronic supplementary material:**

The online version of this article (doi:10.1186/s13045-015-0169-6) contains supplementary material, which is available to authorized users.

## Background

Increased cellular expression and activity of proteasome have been reported in a variety of neoplasms, including hematological, epithelial, and neurological tumors [1–5]. This has led to the proposal of proteasome inhibitors as antineoplastic therapy, with clinically relevant results in some tumors, such as plasma cell myeloma and mantle cell lymphoma [6, 7]. Both epithelial and hemopoietic neoplasms have been associated with increased plasma levels of proteasome which were disease type, stage, and therapy sensitive [8–11]. However, little is known about the intracellular origin and release mechanisms of this increase in plasma proteasome in neoplastic diseases. It remains unclear whether the proteasome in neoplastic cells is passively released during cell lysis and apoptosis, or whether some specific, more regulated, release mechanism exists, which may better account for the selectivity of the changes in plasma proteasome level [9].

Proteasome has been reported in exosomes [12], which are small (50–100 nm) vesicles of endosomal origin that are released by several cell types, including non-pathological blood cells [13, 14] and neoplastic cells [15]. The involvement in proteasome release of other extracellular vesicles, such as plasma-membrane-derived microvesicles, also called ectosomes or microparticles, has recently been suggested [16]. Besides proteasome components, neoplastic cells accumulate excessive amounts of polyubiquitinated proteins [2, 4, 5, 17], the proteasome elective substrate. Limited information is available on the fate of such proteins and whether they are totally degraded inside neoplastic cells or at least in part discharged extracellularly together with proteasome components.

We recently identified and characterized a novel particulate cytoplasmic structure (PaCS) that concentrates polyubiquitinated proteins and proteasome in some epithelial neoplasms and related preneoplastic lesions [5, 7, 17, 18], as well as in two genetic disorders characterized by hematopoietic cell dysplasia and increased risk of leukemia [19, 20]. However, the presence of PaCSs in hematological neoplasms has never been investigated.

Here, we demonstrate that PaCSs accumulate in different forms of myeloid neoplasia in a maturation- and lineage-specific manner. Our findings suggest that a mechanism of PaCSs discharge by leukemic cells contribute to increased plasma proteasome in myeloid neoplasms.

## Results

### PaCSs in hematological neoplasms

**Fig. 1:**
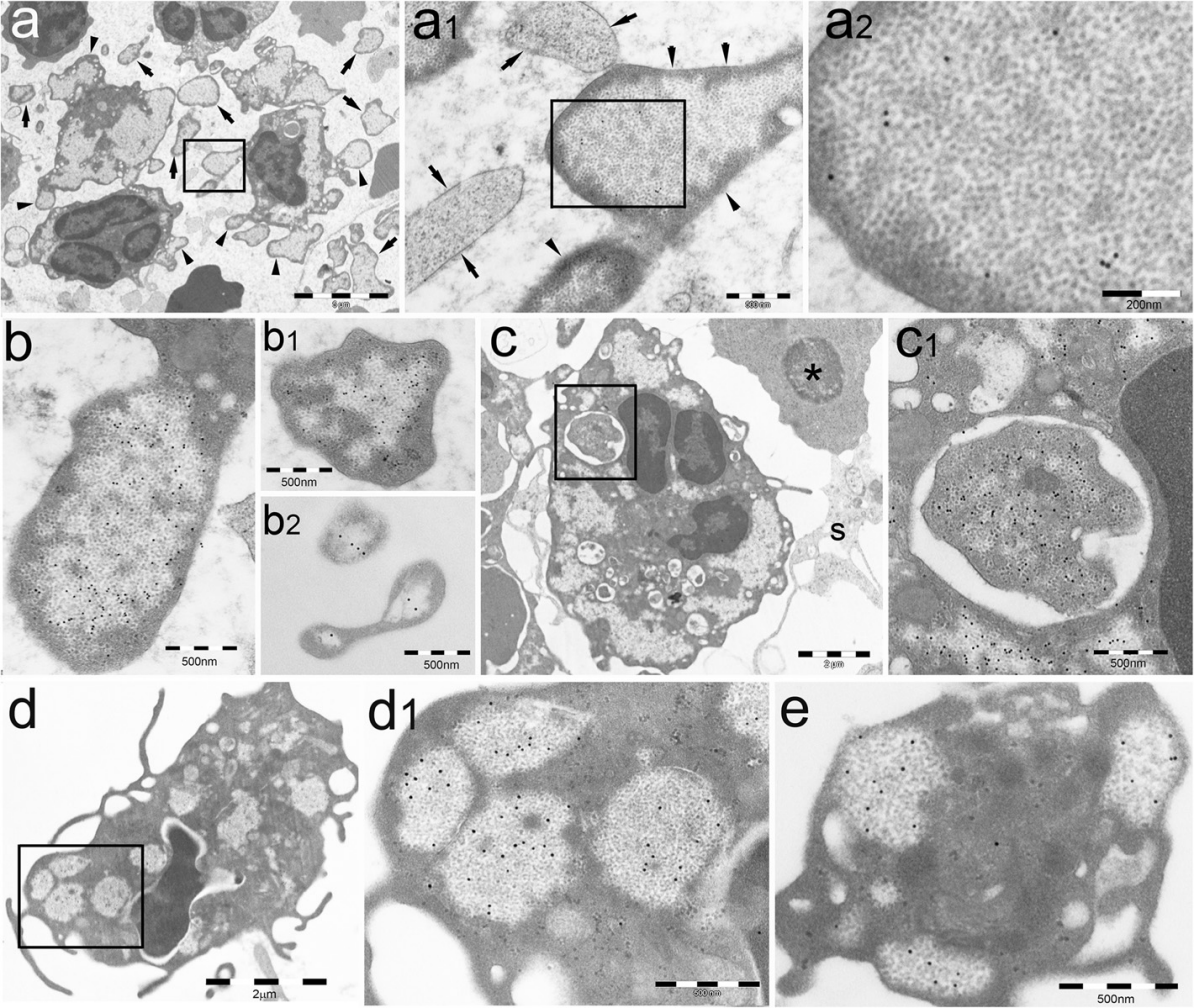
PaCSs are widespread in hematopoietic cells of patients with untreated CML. **a** Several BM cells of the granulocytic lineage display cytoplasmic collections of barrel-like particles, enlarged in **a1** and **a2** to show the particles and their 20S proteasome (β5i subunit) immunogold reactivity. PaCS-filled blebs (*arrowheads*) and cell-detached vesicles (*arrows*) are devoid of cytoplasmic organelles. A cytoskeleton-rich cytoplasmic network (*arrowheads* in **a1**) separates PaCSs from the cytoplasmic membrane; the cytoskeletal network is largely lost in some detached vesicles (*arrow* in **a1**) undergoing degeneration while still preserving proteasome reactivity. **b** A PaCS-filled cellular bleb (**b**) and isolated vesicles (**b1**, **b2**) in BM extracellular space show PaCS-restricted immunogold reactivity for polyubiquitinated proteins (FK1 antibody). **c** A myelocyte shows large, FK1-reactive PaCSs and several FK1-negative autophagic vesicles. The largest vesicle is enlarged in **c1** to show its enveloping double membrane and its unusual storage of PaCS-type particles, as well as FK1-immunoreactive polyubiquitinated proteins comparable with those of adjacent PaCSs; a likely sign of ongoing PaCS autophagy. The *asterisk* indicates an erythroblast showing no PaCSs; *s* indicates stromal cell processes. **d, e** PB granulocytic cell (**d**, enlarged in **d1**) and platelet (**e**) from a CML patient showing PaCSs reactive for polyubiquitinated proteins

**Fig. 2:**
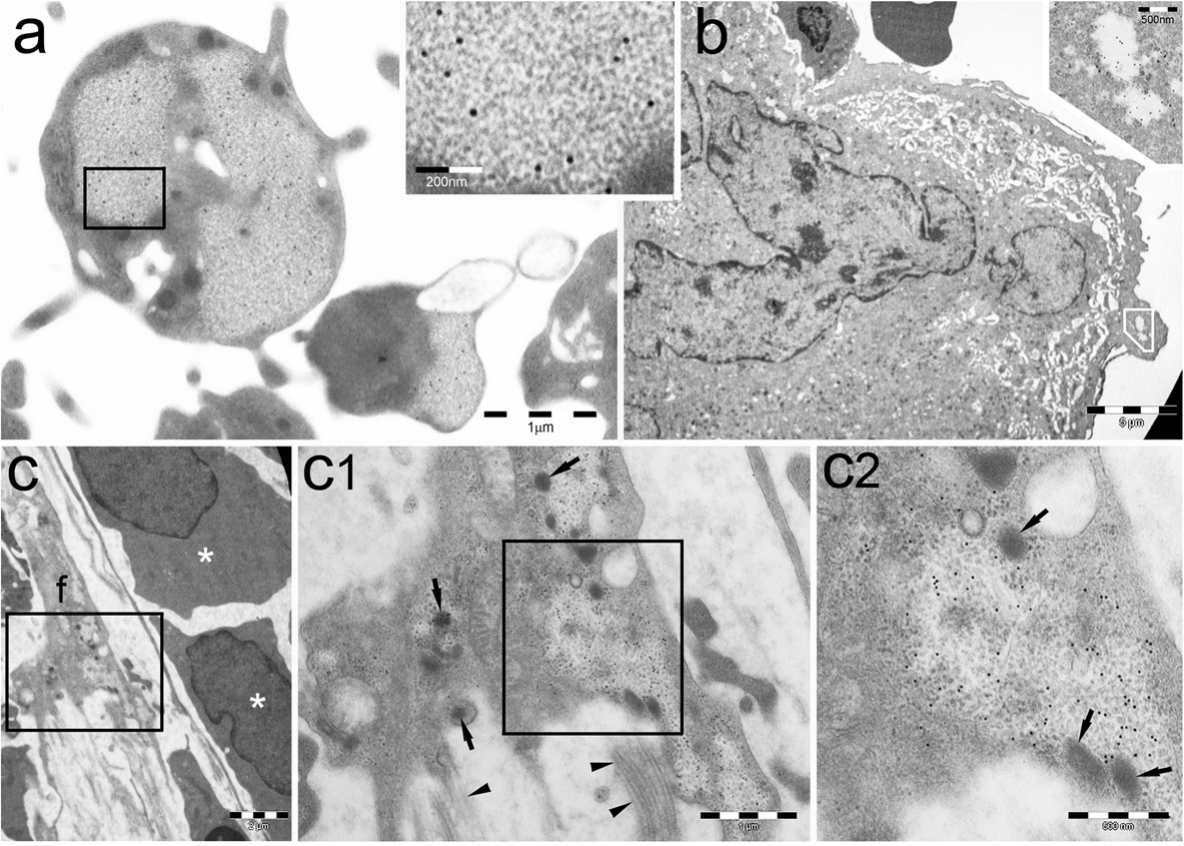
PaCSs are widespread in cells of Philadelphia-negative MPN. **a** PaCSs in PB platelets of a patient with PV, enlarged in the *inset* to show barrel-like particles and FK1 immunoreactivity. **b** BM megakaryocyte with small FK1-reactive peripheral PaCSs, enlarged in the *inset*, from a patient with ET. **c** A fibroblast (*f*) with FK1-positive PaCSs and two intrasinusoidal erythroblasts (*asterisks*) are found in the BM of an individual with PMF. The fibroblast is enlarged in **c1** and **c2** to show the intracellular procollagen granules (*arrows*), juxtafibroblast collagen fibers (*arrowheads*), and FK1-positive PaCSs

Combined ultrastructural and immunogold analysis showed that PaCSs were extensive in bone marrow (BM) of patients with myeloproliferative neoplasms (MPN), including chronic myelogenous leukemia (CML), polycythemia vera (PV), essential thrombocythemia (ET), and primary myelofibrosis (PMF). PaCSs appeared as focal accumulations of barrel-like particles, mostly ~13 nm thick and 13–20 nm long, filling cytoplasmic areas devoid of cytoskeleton or cellular organelles such as mitochondria, endoplasmic reticulum, Golgi complex, and lysosomes, while being often surrounded by ribosomes (Figs. 1 and 2). Similarly to PaCSs previously observed in other disorders [17–20], PaCSs of MPNs showed selective immunogold reactivity for polyubiquitinated proteins and 19S and 20S proteasome, with more than 20-fold higher concentration of gold particles inside PaCSs than in the cytoplasm outside PaCSs. Notably, PaCSs were prominent in granulocytic cells at different stages of maturation (from myelocytes to neutrophil granulocytes) and in mature megakaryocytes (Figs. 1a–c and 2b), whereas no PaCSs were found in erythroblasts, lymphocytes, plasma cells, or macrophages. In the cases of PMF and ET, we occasionally observed PaCSs in BM stromal cells, including fibroblasts, which were increased in the presence of BM fibrosis (Fig. 2c).

**Fig. 3:**
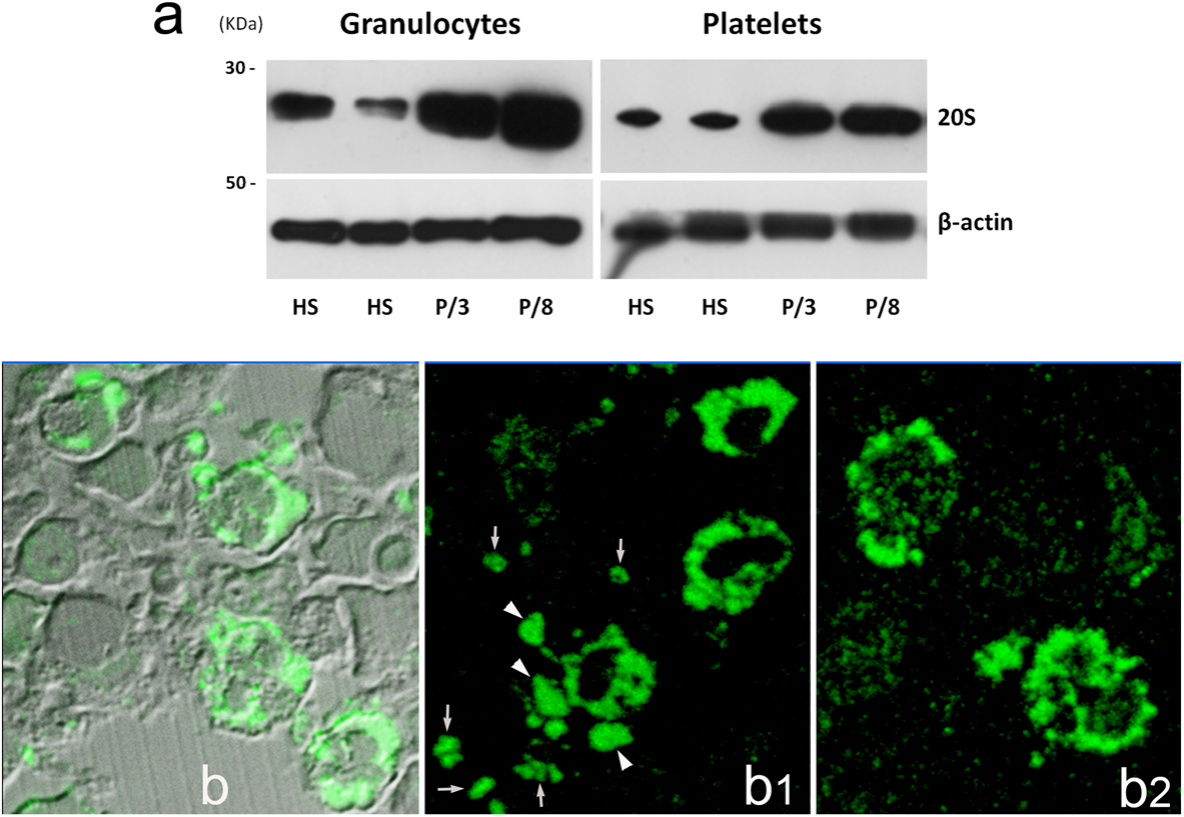
In MPN patients, the proteasome levels are markedly increased in granulocytes and platelet lysates, while immunofluorescence for UPS reveals PaCSs-like structures in BM cells of MPN patients. **a** Accumulation of PaCSs in MPN granulocytes and platelets was associated with markedly increased levels of 20S proteasome in cell lysates. Representative example of Western blot analysis of the 20S proteasome in blood granulocytes and platelet lysates from two healthy subjects (HS) or from patient 3, affected by CML, or patient 8, affected by PV. Granulocyte and platelet lysates were separated on a 12 % SDS/polyacrylamide gel and transferred to nitrocellulose. Membranes were probed with an antibody against 20S proteasome (α6 subunit). Beta-actin was used as equal loading control. **b** Confocal microscopy after immunofluorescence staining for 20S proteasome identifies several cytoplasmic areas with strong 20S signal (*green*) in the BM biopsy of a CML patient; background BM structure is recognized by phase-contrast microscopy. **b1, b2** Two sections from the same specimen show 20S proteasome (**b1**) and polyubiquitinated proteins (**b2**) immunoreactive cytoplasmic areas in CML cells under confocal microscopy; note in **b1** several 20S-positive cell blebs (*arrowheads*) and cell detached vesicles (*arrows*)

For most MPN patients, the analysis was extended to peripheral blood (PB) cells (Table 1). In PB cell preparations, PaCSs were also found extensively in granulocytic cells and in platelets (Figs. 1d, e and 2a), whereas erythrocytes, lymphocytes, and monocytes presented no PaCSs. Consistent with these findings, Western blot analysis demonstrated that protein extracts of both granulocytes and platelets from patients with MPN presented markedly increased levels of 20S proteasome with respect to healthy subjects (Fig. 3a). The PB samples from the two patients with relapsed CML presented PaCSs with distribution, morphology, and immunoreactivity pattern similar to those of untreated CML subjects.

**Table 1:** Study population and samples that were investigated for the presence of PaCSs

Patient	Sex/age	Diagnosis^a^	Disease status	Investigated sample
1	M/64	CML, *BCR/ABL* positive	U	BM, PB
2	M/38	CML, *BCR/ABL* positive	U	BM
3	F/24	CML, *BCR/ABL* positive	U	PB
4	F/79	CML, *BCR/ABL* positive	U	PB
5	F/78	CML, *BCR/ABL* positive	U	PB
6	F/65	CML, *BCR/ABL* positive	U	PB
7	F/79	CML, *BCR/ABL* positive	R	PB
8	F/74	CML, *BCR/ABL* positive	R	PB
9	F/45	ET	U	BM, PB
10	M/56	PV	U	BM, PB
11	M/79	PV	U	BM, PB
12	M/68	PMF	U	BM, PB
13	F/57	PMF	U	BM
14	F/43	AML not otherwise specified, acute myelomonocytic leukemia (A mutation of NPM1 and internal tandem duplication of FLT3)	U	BM
15	M/64	AML not otherwise specified, acute myelomonocytic leukemia (A mutation of NPM1)	U	BM
16	F/64	AML not otherwise specified, acute monoblastic/monocytic leukemia (A mutation of NPM1)	U	BM
17	F/48	AML not otherwise specified, AML with minimal differentiation (A mutation of NPM1)	U	BM
18	M/64	AML not otherwise specified, AML with minimal differentiation (A mutation of NPM1)	U	BM
19	F/18	AML not otherwise specified, AML with minimal differentiation	U	BM
20	M/23	AML not otherwise specified, acute megakaryoblastic leukemia	U	BM
21	M/35	AML not otherwise specified, AML with minimal differentiation (internal tandem duplication of FLT3)	R	BM
22	M/74	MDS, refractory cytopenia with multilineage dysplasia	U	BM, PB
23	M/69	MDS, refractory cytopenia with multilineage dysplasia	U	BM, PB
24	F/35	MDS, refractory anemia with excess blasts	U	BM, PB
25	M/75	MDS, refractory cytopenia with multilineage dysplasia	U	PB
26	M/75	MDS, refractory cytopenia with multilineage dysplasia	U	PB
27	M/52	MDS, refractory anemia with excess blasts	U	PB
28	M/4	MDS, childhood MDS	U	BM
29	F/37	MDS, refractory thrombocytopenia	U	BM, PB
30	M/49	Hairy cell leukemia	U	BM
31	F/50	Chronic lymphocytic leukemia	U	BM, PB
32	M/50	Plasma cell myeloma	U	BM
33	M/80	Plasma cell myeloma	U	BM

^a^According to WHO Classification of Tumors of Hematopoietic and Lymphoid Tissue. Lyon, France: IARC, 2008

*U* untreated, *R* relapsed, *BM* bone marrow iliac biopsy, *PB* cell preparations of peripheral blood granulocytes, mononuclear cells, and platelets

Previous correlative confocal and electron microscopy demonstrated that PaCSs are recognized also by immunofluorescence for 19S, 20S, or polyubiquitinated proteins on osmium-fixed semithin resin sections [5, 19]. As expected, immunofluorescence analysis confirmed that BM cells of patients with MPNs presented cytoplasmic areas with strong immunoreactivity to UPS components (Fig. 3b).

**Fig. 4:**
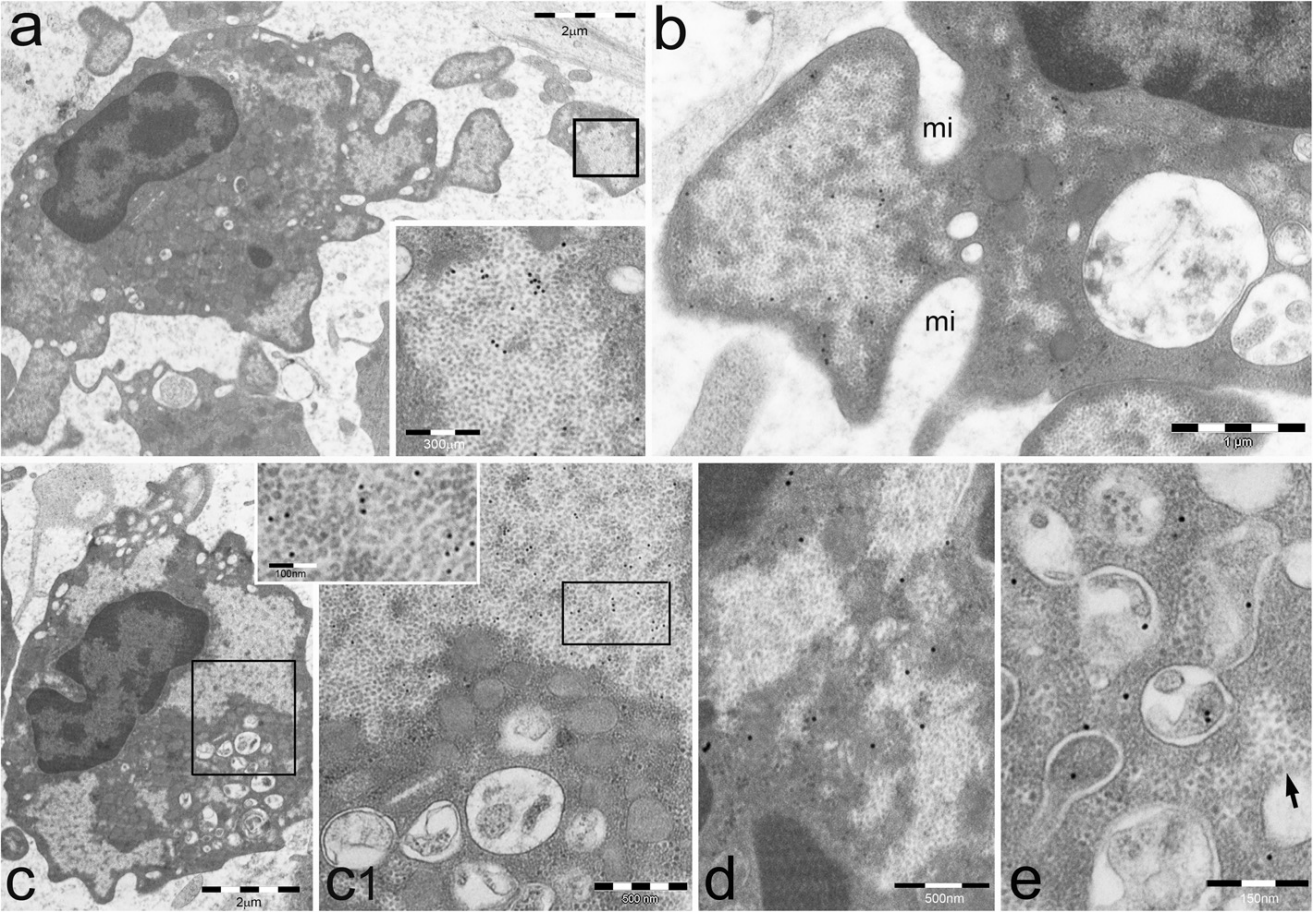
PaCSs present selective immunoreactivity for chaperone molecules. **a–e** BM MPN cells show selective PaCS immunoreactivity for Hsp40 (**a**), Hsp70 (**b**), and Hsp90 (**c**, **c1**, and *inset*), although not for Bag6 (**d**). Autophagic vesicles remain unreactive to Hsp70 (**b**) and Hsp90 (**c**, **c1**), while reacting with LC3A antibody (**e**), where an adjacent small PaCS (*arrow*) does not react. *mi* plasma membrane invaginations in the process of severing PaCS-filled blebs from their cell of origin

A prominent feature of PaCS-positive BM cells from MPN patients was the presence of pleomorphic PaCS-filled and organelle-free cytoplasmic membrane blebs of varying size and shape. These blebs apparently released PaCS-filled round to ovoid vesicles (0.1–1 μm in diameter) that were fully detached from the cytoplasm and freely floating in the BM intercellular spaces (Figs. 1a, b and 4a, b). In some cells with PaCSs, many autophagic vesicles, characteristically enveloped by double membranes, were also seen in the cytoplasm (Fig. 1c–e). A minority of such vesicles stored PaCS-type particles and contained polyubiquitinated proteins and proteasome immunoreactivity, which strongly suggests that PaCSs were partly involved in the autophagic process (Fig. 1c). Known ultrastructural signs of apoptosis [19, 21], such as chromatin compaction, nuclear membrane loss, and dense and homogeneous cytoplasm with no recognizable organelles were observed only occasionally in MPN cells, irrespectively of the presence of PaCS, blebs/ectosomes, or autophagic vesicles.

To gain insight into the respective role of PaCSs and autophagic vesicles, we performed in MPN cells cytochemical assays for proteins known to be associated with UPS function or autophagy. Hsp40, Hsp70, and Hps90 chaperones were selectively concentrated inside PaCSs (Fig. 4a–c), while the co-chaperone Bag 6 had sparse cytoplasmic reactivity, without preferential concentration in PaCSs (Fig. 4d). Autophagic vesicles showed reactivity for LC3A protein, a known autophagy marker [22], to which PaCSs showed no reactivity (Fig. 4e).

**Fig. 5:**
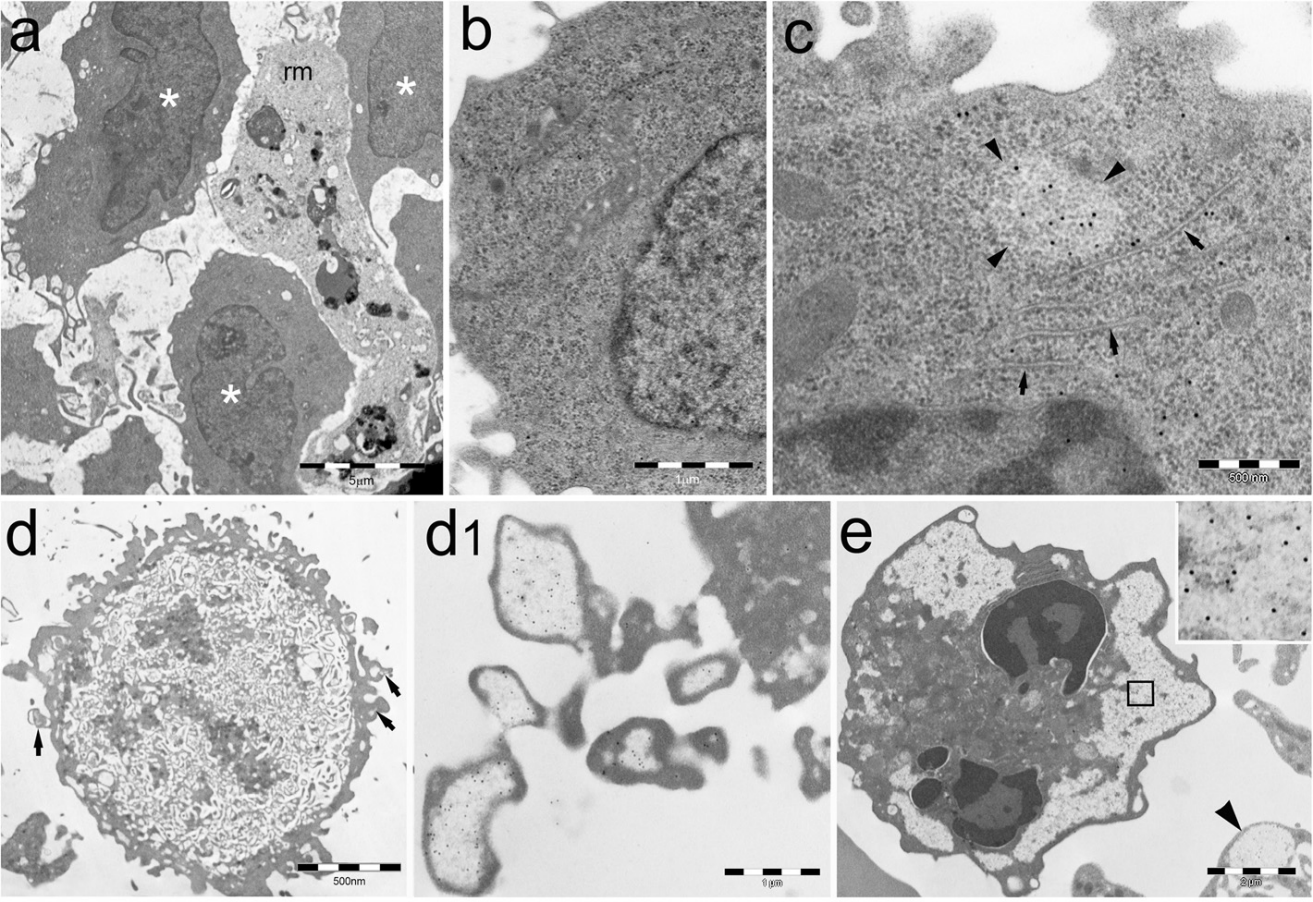
PaCSs are poorly represented in AML whereas are present in MDS cells. **a–c** BM of AML patients. No PaCSs are found in blast cells shown in (**a**) (*asterisk*) and (**b**), while a small FK1-reactive PaCS (*arrowheads*) is being formed in a ribosome-rich area of another blast (**c**), also showing focal differentiation of endoplasmic reticulum (*arrows*). *rm* reactive macrophage. **d** BM micromegakaryocyte of a patient with MDS (refractory anemia with multilineage dysplasia) showing small peripheral PaCSs (*arrows*), some of which enter into blebs and free vesicles (**d1**). **e** PB neutrophil granulocyte of a patient with MDS (refractory anemia with multilineage dysplasia) with several PaCSs, one of which is enlarged in the *inset* to recognize FK1 immunogold reactivity. Note in the lower right corner (*arrowhead*) a PaCS in an adjacent platelet

Unlike MPN cells, BM blasts of the 8 patients with acute myeloid leukemia (AML) had no PaCSs or only a few small PaCSs, and they had no autophagic vesicles or PaCS-filled blebs (Fig. 5a–c) In BM biopsies of AML patients, only the cells with morphological features of granulocytic precursors or megakaryocytes showed PaCSs. The findings on the relapsed AML patient were similar to those of the 7 untreated cases. Among the 8 patients with myelodysplastic syndromes (MDS), PaCSs were found in BM cells of the granulocytic and megakaryocytic lineages, as well as in PB granulocytes and platelets of all the analyzed patients (Fig. 5d, e). A notable exception was represented by BM granulocytic and megakaryocytic precursors with most prominent signs of cytoplasmic immaturity (abundant ribosomes, paucity of cytoplasmic organelles, and reduced specific secretory granules), which usually lacked or showed only occasional small PaCSs. Similarly, blast cells of the patients with refractory anemia with excess blasts had no PaCSs (data not shown). These findings suggest that, within the myeloid neoplastic clones, PaCSs are widespread in cells with preserved, although aberrant, maturation toward granulocytic or megakaryocytic lineages, whereas they do not develop in cells with maturation arrest at the early stage of blast cells or of very immature dysplastic precursor. PaCSs of MDS presented the same immunoreactivity pattern of those observed in MPN; in particular, PaCSs of MDS patients also showed selective concentration of Hsp40, Hsp70, and Hps90 (data not shown). Moreover, the presence of PaCS-filled cytoplasmic membrane blebs, associated with PaCS-filled vesicles freely floating in the intercellular space, has been frequently observed also in BM of the MDS subjects (Fig. 5d).

There were no PaCSs in BM biopsies of 4 patients with chronic B-cell leukemia or plasma cell myeloma (Additional file 1: Figure S1). Myeloma plasma cells showed sparse proteasome immunoreactivity in close association with the extensively developed rough endoplasmic reticulum (RER) cisternae, some of which were dilated and filled with compact, amorphous material occasionally forming Russel or Dutcher bodies (Additional file 1: Figure S1e, f).

### PaCSs in non-neoplastic BM and PB

Ultrastructural analysis of non-neoplastic BM biopsies from 6 individuals, combined with immunogold labeling for polyubiquitinated proteins and 20S and 19S proteasome, showed no PaCSs in hematopoietic or stromal cells (Additional file 1: Figure S2a–e). There were occasional small PaCSs in cells of the granulocytic or megakaryocytic lineages inside areas with increased macrophages, eosinophils, plasma cells, or lymphocytes, likely due to focal inflammation. There was increased accumulation of polyubiquitinated proteins in the cytoplasm of some mature BM granulocytes, sometimes concentrated in cytoplasmic blebs; particularly in minute clear, non-particulate “areolae,” as previously described [19] (Additional file 1: Figure S2f). PB from healthy volunteers showed small scanty PaCSs in 10–20 % of granulocytes and platelets (Table 2; Additional file 1: Figure S2g, h), consistent with previous observations on PB granulocytes and platelets obtained from healthy subjects [19, 20].

**Table 2:** Morphometric features of PaCSs in granulocytes and platelets of four CML patients and three healthy subjects

	Cells with PaCSs (%)	PaCS area/total cytoplasm area (%)	Number of PaCSs/cytoplasm area unit (n/nm^2^ × 10^8^)	Number of PaCSs/cell
Granulocytes				
CML	67.8 ± 9.1**	7.1 ± 1.9**	53.8 ± 28.4*	8.2 ± 4.5*
Healthy subjects	15.3 ± 12.8	1.1 ± 0.5	5.1 ± 3.9	0.7 ± 0.5
Platelets				
CML	56.5 ± 5.0***	7.6 ± 1.2***	47.4 ± 18.7*	0.9 ± 0.3*
Healthy subjects	18.2 ± 4.8	0.9 ± 0.3	11.5 ± 7.6	0.2 ± 0.1

Data are presented as means ± SD

*P < 0.05 vs. healthy subjects

**P < 0.01 vs. healthy subjects

***P < 0.001 vs. healthy subjects

### Quantification of PaCSs in PB cells from CML patients and healthy controls

To confirm and quantify the increased presence of PaCSs as a feature of hematopoietic cells in MPN, we performed software-assisted image analysis of electron microscopy preparations of PB granulocytes and platelets from 4 consecutive CML patients and three healthy subjects. The morphometric features of PaCSs are reported in Table 2. In particular, the mean area of cytoplasm occupied by PaCSs was 6.5-fold higher in CML than control granulocytes (*P* < 0.01) and 8.4-fold higher in CML than control platelets (*P* < 0.001). For both granulocytes and platelets, the increased presence of PaCSs in CML patients compared with healthy subjects resulted from a higher percentage of cells with PaCSs and a higher number of PaCSs per cell.

### Plasma proteasome levels in patients with MPN, MDS, and healthy controls

Plasma proteasome levels were measured in 6 patients with CML (patients 3–8 in Table 1), 4 subjects with PV or PMF (patients 10–13), 6 individuals with MDS (22–27), and 10 healthy subjects. Compared to healthy individuals, proteasome levels were significantly higher in CML patients (mean ± standard deviation: CML 12.2 ± 7.6 μg/mL vs. healthy subjects 3.3 ± 2.0 μg/mL, *P* < 0.01), in patients with *BCR/ABL*-negative MPN (14.6 ± 6.7 μg/mL, *P* < 0.001 vs. healthy controls), as well as in MDS individuals (17.5 ± 14.3 μg/mL, *P* < 0.01 vs. healthy controls).

## Discussion

This study showed that PaCSs, the recently identified cytoplasmic structures rich in proteasomes and polyubiquitinated proteins [18, 23], were expressed in myeloid neoplasia. In particular, PaCSs were widespread in BM and PB cells of MPN patients. In these cases, PaCSs were restricted to cells of the granulocytic lineage, megakaryocytes, and platelets but were absent in immature blasts as well as in erythroid cells. Among AML and MDS patients, PaCSs were absent or scarce and small in blast cells, while they were detected in cells undergoing granulocytic or megakaryocytic maturation. No PaCSs were found in B-cell chronic leukemia, plasma cell myeloma, or non-neoplastic BM. Thus, as already observed in fetal tissues [17] and solid neoplasms [5], in myeloid neoplasms, the development and accumulation of PaCSs appears associated with differentiation along specific hematopoietic lineages. These may be instrumental in allowing certain cytokines or growth factors to induce PaCSs through lineage-specific receptors or signalling pathways [23]. This mechanism of lineage specificity may explain why neoplasms with lymphoid or plasma cell differentiation are not associated with development of these cytoplasmic structures.

PaCSs arise from rearrangement of cytosolic UPS components from their usually diffuse pattern in most normal adult cells [17, 24] to a focal concentration in distinctive, particle-rich areas [18, 23]. The driving factors, mechanisms, and biological significance of this change remain largely unknown. However, PaCS-developing cells have several points in common, which may include an ongoing differentiation process coupled with enhanced proliferation (as found in both fetal and neoplastic cells) [17], chronic infection [18], or even stimulation by cell-specific growth factors and interleukins [23]. All such conditions are known to enhance cellular metabolism and to require high UPS function [25–27], thus increasing the rate of proteasome-dependent degradation of misfolded proteins [28]. This is a potential cause of UPS stress and impaired protein homeostasis, as frequently found in neoplastic cells [1, 2, 4, 29].

A prominent and constant finding of PaCSs is accumulation of polyubiquitinated proteins [5, 17, 18], which by itself would suggest proteasome malfunction [30]. However, we also found in PaCSs selective concentration of proteasome proteins and activity [18, 23], in keeping with the increase in both polyubiquitinated proteins and proteasome protein and activity reported in neoplastic cell lines and primary neoplastic tissues [1, 3, 4, 29]. A relative insufficiency of proteasome activity in relation to an excessively high rate of production of polyubiquitinated (misfolded) proteins may explain these findings, given the evidence for increased activity of E1 ubiquitin-activating enzyme, which is crucial for protein ubiquitination, especially in hemopoietic neoplasms [29].

The selective high concentration of multiple chaperone molecules in PaCSs is of high biological interest. Hsp40 and Hsp70 are known to interact with newly synthesized proteins as they come off the ribosomes and to enable their appropriate folding. When folding and repair of misfolded and denatured proteins fail, Hsp70 promotes their ubiquitination and proteasome-dependent degradation [31]. However, Hsp90 can be recruited to the Hsp70/Hsp40/misfolded protein complex with resulting protein stabilization and prevention of its proteasome degradation [32–34]. This mechanism involves many mutated or overexpressed receptor kinases, signaling peptides, and transcription factors implicated in cancer, which are chaperoned by Hsp90 in a way that they can bind their ligand and undergo persistent activation. In this way, the Hsp90/oncoprotein complex activates cellular growth signals, blocks anti-growth signals, sustains angiogenesis, and evades apoptosis [34–36]. Among targets specifically activated by Hsp90 in myeloid neoplasms are the BCR-ABL kinase and several receptor tyrosine kinases, including TrkA, which stimulate tumor cell proliferation and survival [34, 37]. Thus, the finding that PaCSs selectively concentrate Hsp90, Hsp70, and Hsp40 suggests that these structures are involved in the formation and/or accumulation of the multi-chaperone complexes with high affinity for client oncoproteins that play a key role in proliferation of neoplastic clones [33–35]. The Hsp90 inhibitors have much higher affinity for Hsp90 bound to these complexes than for uncomplexed Hsp90, resulting in oncoprotein destabilization and UPS-mediated degradation [33, 34]. In this context, we hypothesize that the high presence of PaCSs in certain tumor cells could be associated with a high sensitivity to Hsp90 inhibitors.

Our findings suggest that when excess PaCSs accumulate in the cytoplasm, as in the case of MPN, the cells can activate two distinct processes to remove high levels of potentially toxic, polyubiquitinated misfolded proteins, namely: autophagic degradation, or extracellular discharge through PaCS-filled blebs, generating cytoplasmic vesicles (ectosomes). Autophagy was a consistent finding only in neoplastic myeloid cells, while PaCSs-filled, UPS-rich blebs and vesicles were released frequently also by non-hematological neoplastic cells [17] and by non-neoplastic, especially immunocompetent cells [23]. This may represent a sort of non-canonical secretory discharge system, shown to operate in intercellular communication among neoplastic and immunocompetent cells [13, 15, 38]. In particular, the significant increase in plasma proteasome content in CML patients who also had increased PaCSs as well as increased proteasome content in their leukemic cells suggests that release of UPS-rich PaCS content is a source of plasma proteasome. This fits with the previously reported higher plasma proteasome content of myeloid compared to lymphoid leukemia or plasma cell myeloma [9]. Although in principle, tumor cell lysis or apoptosis may explain in part such increased proteasome plasma levels, parallel measurement of lactate dehydrogenase, and other biological markers of cell damage fails to account for changes in proteasome levels [8, 9]. We failed to detect signs of apoptosis in most PaCS-storing cells, with or without associated blebs or vesicles. This leaves room for a more specific, cell-type-restricted mechanism such as plasma membrane vesicle release, which has been found to be selectively regulated in several experiments [14]. In principle, this mechanism of PaCSs discharge through ectosomal microvesicle release may also contribute to the non-canonical, possibly exosome-mediated Hsp90 secretion that occurs in neoplastic cells and enhances their invasive potential [39, 40].

## Conclusions

This study demonstrates for the first time that PaCSs accumulate in cells of myeloid neoplasms; in particular, these structures were widespread in granulocytic cells, megakaryocytes, and platelets of patients with MPN. PaCSs development was associated with excess accumulation of proteasome and polyubiquitinated proteins, likely indicating impairment of the UPS-dependent protein homeostasis. The selective concentration in PaCSs of Hsp40, Hsp70, and Hsp90 suggests a role for these structures in the formation of the multi-chaperone complexes involved in Hsp90-mediated leukemogenesis. In addition, a microvesicle release mechanism of PaCSs discharge by leukemic cells has been observed, which may contribute to the increased plasma proteasome found in MPN and MDS. Definition of the role of PaCSs in myeloid neoplasms is worth of further investigation, as it may open new prospects in the comprehension of pathophysiology of these disorders.

## Patients and methods

### Patients

This study included 29 patients affected by different forms of myeloid neoplasms. Four patients with chronic B-cell leukemia or plasma cell myeloma were also investigated. Table 1 summarizes the baseline characteristics of the patients and the samples that were investigated for the presence of PaCSs. Twenty-six patients were investigated at a time when they were free from any specific treatment for the hematological disease. Three patients were studied at relapse: they were two subjects with CML relapsed after tyrosine kinase inhibitors and one AML patient relapsed after conventional chemotherapy. In all the cases, BM biopsy was performed as part of the diagnostic workup. BM biopsies from six individuals without any apparent BM pathology were also investigated. In two cases, BM biopsy was taken from the femoral head that was removed during surgery for hip prosthesis in patients with osteoarthritis. In four cases, iliac BM biopsies were taken as part of the staging procedure for non-Hodgkin’s or Hodgkin’s lymphoma and were free from the lymphoproliferative disease after histological and immunophenotypic BM examination. Control PB samples were obtained from healthy volunteers. Granulocyte, mononuclear cell, and platelet fractions were separated from PB samples as previously reported [20]. The study subjects or their legal guardians gave informed consent for this study, which was approved by the Institutional Review Board of the IRCCS Policlinico San Matteo Foundation, Pavia, Italy.

### Electron microscopy and immunogold analysis

BM biopsies or PB cell preparations were fixed immediately after sampling in 2.5 % glutaraldehyde and 2 % paraformaldehyde in pH 7.3 cacodylate buffer for 4 h at 4 °C, followed by post-fixation in 1.5 % osmium tetroxide for 1 h at room temperature, and Epon-Araldite embedding [18]. Ultrathin sections (~70 nm thick) were cut from the resin blocks and stained with uranyl acetate/lead citrate for conventional electron microscopy or underwent immunogold staining as previously reported [17, 23]. Sections were incubated in 10 % normal goat or bovine serum (Dako, Glostrup, Denmark) for 1 h at room temperature, followed by incubation with primary antibodies (as specified below) and secondary anti-mouse, anti-rabbit, or anti-goat IgG or IgM labeled with 6-, 10-, 15-, or 20-nm gold particles (Aurion ImmunoGold Reagents, Wageningen, Netherlands; or BB International, Cardiff, UK). Sections were counterstained with uranyl acetate/lead citrate. Specimens were analyzed by a Jeol JEM-1200 EX II (JEOL Ltd, Tokyo, Japan) transmission electron microscope equipped with an Olympus Mega View III CCD camera (Tokyo, Japan). The following primary antibodies were used: mouse FK1 monoclonal against polyubiquitinated proteins [41] (Enzo Life Sciences International, Plymouth Meeting, PA, USA); rabbit polyclonal against the 20S proteasome, αβ or β5i subunits (Calbiochem, La Jolla, CA, USA); rabbit polyclonal against 19S proteasome, S2 subunit (Calbiochem); rabbit polyclonal against Hsp40 (LS Bio, Seattle, WA, USA); goat polyclonal against Hsp70 (Santa Cruz Biotechnology, Santa Cruz, CA, USA); mouse 4F3-E8 monoclonal against Hsp90 (Novus Biologicals, Littleton, CO, USA); rabbit polyclonal against BAG6 (Santa Cruz Biotechnology); rabbit polyclonal against LC3A (Abgent, San Diego, CA, USA). The specificity of immunogold labeling was evaluated by omitting the specific antibodies in the first layer of the procedure or by substituting them with non-immune IgG or IgM (Santa Cruz Biotechnology) or unrelated antibodies.

### Software-assisted image analysis

Image analysis was carried out on PB granulocyte and platelet electron microscopy preparations from 4 consecutive patients affected by CML (patients 3–6 in Table 1) and three healthy subjects. Patient samples were collected and processed for TEM simultaneously with control samples in three different experiments. Sections were analyzed after immunogold staining with the FK1 antibody against polyubiquitinated proteins. Images were taken at a 25,000× magnification, and a mean number of 25.7 ± 1.8 granulocyte sections and 65 ± 10.2 platelet sections were analyzed for each subject. Morphometric observations were carried out by the ITEM soft imaging system (Olympus Soft Imaging Solutions GmbH). The following parameters were directly measured: presence, number, and area of each individual PaCS; area of the cytoplasm of each analyzed cell; and number of immunogold particles inside PaCSs and in the cytoplasm outside PaCSs. Data were expressed as means ± SD. Statistical analysis was performed by two-tailed Student’s *t* test.

### Immunofluorescence analysis

Semithin (about 0.1 μm) ultramicrotomic sections were obtained from the resin blocks and used for immunofluorescence confocal and phase contrast microscopy. After washing with PBS, sections were incubated overnight with primary and then with secondary antibodies as described [23]. Primary antibodies against 19S, 20S, and polyubiquitinated proteins are the same used for electron microscopy. Secondary antibodies were Alexa Fluor 488-conjugated anti-mouse or anti-rabbit IgG (Life technologies, Paisley, UK). Specimens were analyzed by a TCS SP5II confocal laser scanning microscope equipped with PL APO 40x/1.25 NA and 63/1.40 NA oil-immersion objectives (Leica, Heidelberg, Germany).

### Western blotting analysis

Granulocytes and platelets lysates were prepared and dissociated as previously described [20].

Equal amounts of samples were separated in a 12 % polyacrylamide SDS-PAGE gel and transferred to nitrocellulose (Bio-Rad, Hercules, CA, USA). After blocking with 5 % not-fat milk, membranes were probed with mouse monoclonal MCP against proteasome 20S α6 subunit (Enzo Life Sciences) or mouse monoclonal AC-15 against β-actin (Sigma, St. Louis, MO, USA). Membranes were then incubated with horseradish peroxidase-conjugated anti-mouse antibody, and protein bands were visualized by an enhanced chemiluminescence method (GE Healthcare, Waukesha, WI, USA).

### ELISA of plasma proteasome levels

Plasma samples were obtained by centrifugation of whole blood anti-coagulated with citrate for 15 min at 1000 *g* and maintained at –20 °C until analysis. The proteasome levels were measured by the 20S/26S Proteasome ELISA Kit (Enzo Life Sciences). Plasma samples were diluted 1:8 with the ELISA buffer provided by the kit and assayed in duplicate. Optical density was read at 450 nm on a microplate reader (Model 680; Bio-Rad, Hercules, CA, USA). Data are representative of three separate measurements. Statistical analysis was performed by two-tailed Student’s *t* test.

## Additional file

Additional file 1:
**Supplementary figures.** **Figure S1.** No PaCSs are found in chronic B-cell leukemia and plasma cell myeloma. **Figure S2.** PaCSs are not found in non-neoplastic BM cells, whereas some PaCSs are identified in PB granulocytes and platelets from healthy subjects.
