# Supplementary material for: Particulate cytoplasmic structures with high concentration of ubiquitin-proteasome accumulate in myeloid neoplasms
Source: J Hematol Oncol. 2015 Jun 18;8:71. doi: 10.1186/s13045-015-0169-6 (PMC4473848; doi:10.1186/s13045-015-0169-6)
Supplement: Additional file 1: — Supplementary figures. Figure S1. No PaCSs are found in chronic B-cell leukemia and plasma cell myeloma. Figure S2. PaCSs are not found in non-neoplastic BM cells, whereas some PaCSs are identified in PB granulocytes and platelets from healthy subjects. [file 13045_2015_169_MOESM1_ESM.pdf]

ADDITIONAL DOCUMENTATION

Pecci et al., Particulate cytoplasmic structures with high concentration of ubiquitin-proteasome accumulate in myeloid neoplasms

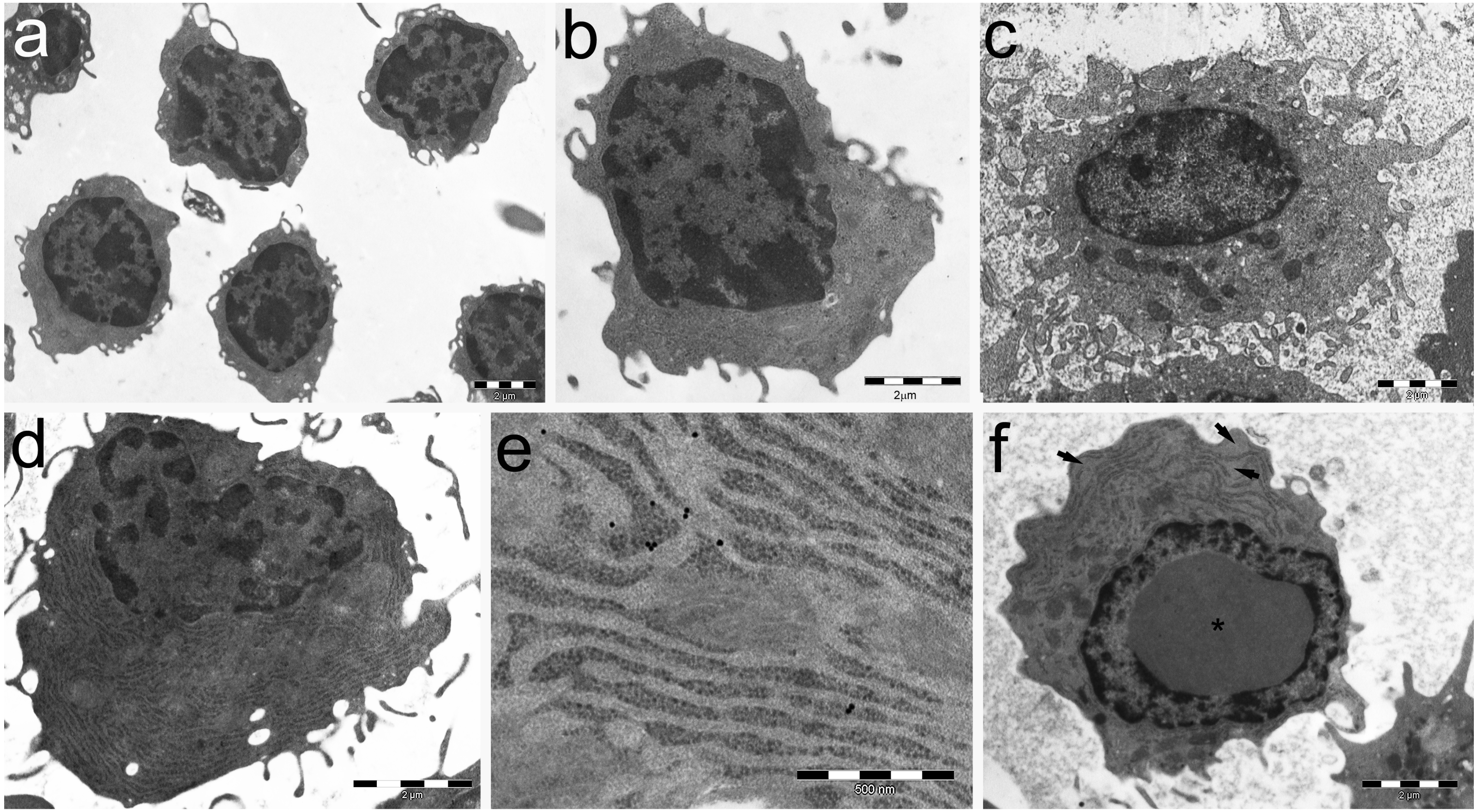

Additional Figure 1. No PaCSs are found in chronic B cell leukemia and plasma cell myeloma. (a-f): Representative examples of neoplastic cells in BM samples from patients with chronic lymphocytic leukemia (a, b), hairy cell leukemia (c) or plasma cell myeloma (d–f). In e, the sparse proteasome 20S immunoreactivity in close correlation with extensively developed RER, and in f, the amorphous (immunoglobulin) deposits filling some RER cisternae (arrows), as well as a nuclear invagination (Dutcher body, *).

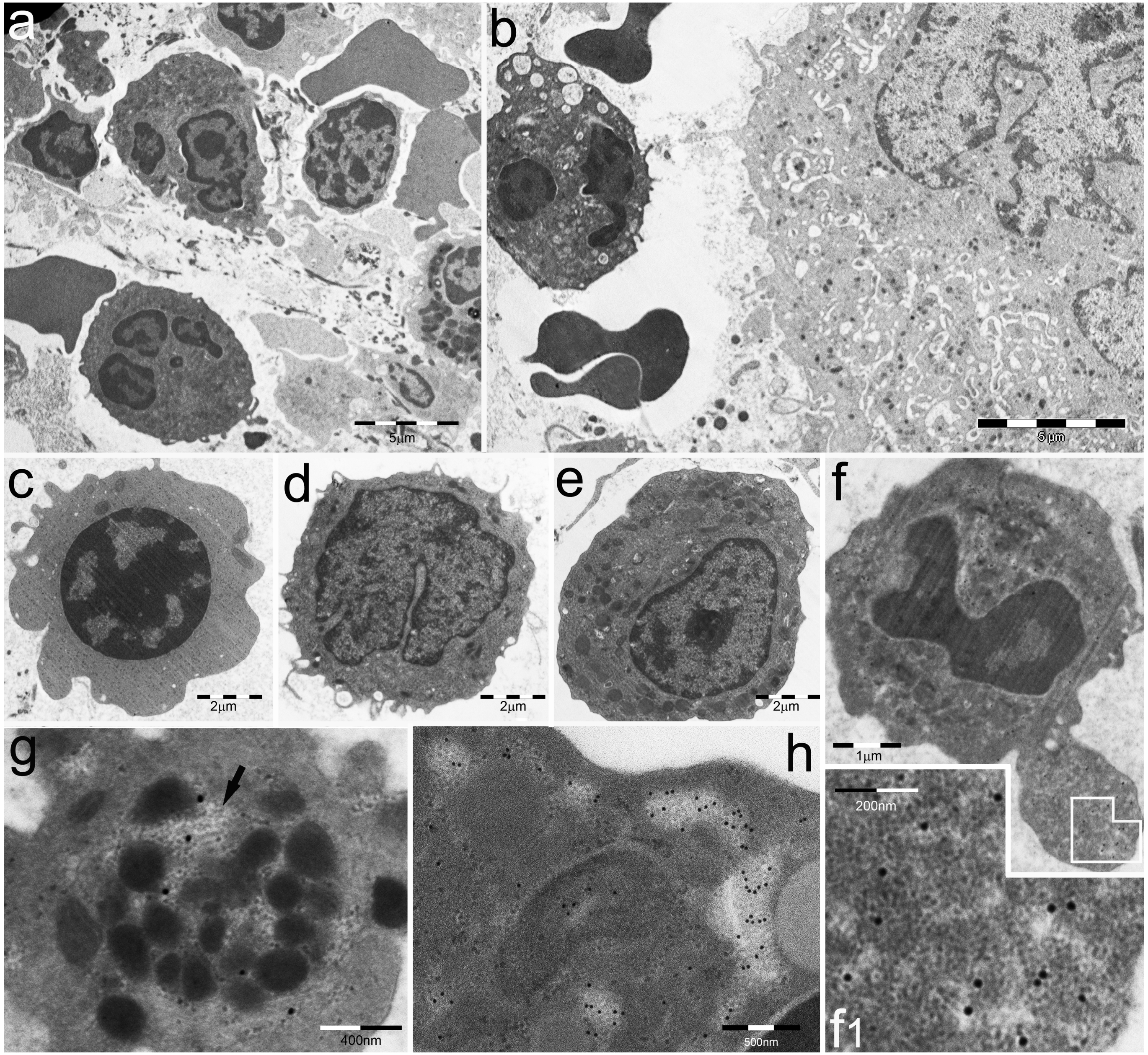
a
5μm
b
5μm
c
2μm
d
2μm
e
2μm
f
1μm
200nm
f1
g
400nm
h
500nm

Additional Figure 2. PaCSs are not found in non-neoplastic BM cells, whereas some PaCSs are identified in PB granulocytes and platelets from healthy subjects. (a-f): Representative examples of BM cells from healthy individuals, including several granulocytic precursors (a), megakaryocyte, granulocyte and erythrocytes (b), erythroblast (c), monocyte (d), another granulocytic precursor cell (e), and a differentiated neutrophil granulocyte (f). The latter forms an organelle-free cytoplasmic bleb selectively enriched in polyubiquitinated proteins dispersed in the ribosome-rich cytoplasm, often inside minute clear areolae (f1). **(g, h):** PB cells from control subjects with a few FK1-reactive PaCSs are seen in a platelet (g) and a neutrophil granulocyte (h).
